# Synaptic Defects in the Spinal and Neuromuscular Circuitry in a Mouse Model of Spinal Muscular Atrophy

**DOI:** 10.1371/journal.pone.0015457

**Published:** 2010-11-11

**Authors:** Karen K. Y. Ling, Ming-Yi Lin, Brian Zingg, Zhihua Feng, Chien-Ping Ko

**Affiliations:** Section of Neurobiology, Department of Biological Sciences, University of Southern California, Los Angeles, California, United States of America; Medical College of Georgia, United States of America

## Abstract

Spinal muscular atrophy (SMA) is a major genetic cause of death in childhood characterized by marked muscle weakness. To investigate mechanisms underlying motor impairment in SMA, we examined the spinal and neuromuscular circuitry governing hindlimb ambulatory behavior in SMA model mice (SMNΔ7). In the neuromuscular circuitry, we found that nearly all neuromuscular junctions (NMJs) in hindlimb muscles of SMNΔ7 mice remained fully innervated at the disease end stage and were capable of eliciting muscle contraction, despite a modest reduction in quantal content. In the spinal circuitry, we observed a ∼28% loss of synapses onto spinal motoneurons in the lateral column of lumbar segments 3–5, and a significant reduction in proprioceptive sensory neurons, which may contribute to the 50% reduction in vesicular glutamate transporter 1(VGLUT1)-positive synapses onto SMNΔ7 motoneurons. In addition, there was an increase in the association of activated microglia with SMNΔ7 motoneurons. Together, our results present a novel concept that synaptic defects occur at multiple levels of the spinal and neuromuscular circuitry in SMNΔ7 mice, and that proprioceptive spinal synapses could be a potential target for SMA therapy.

## Introduction

Spinal muscular atrophy (SMA), a leading genetic cause of infant mortality, is an autosomal recessive motoneuron disease characterized by spinal motoneuron loss, muscle atrophy and motor impairment [1], [2]. This disease is caused by deletion or mutation of the *survival of motor neuron 1* (*SMN1*) gene and low expression of the SMN protein derived from the closely-related *SMN2* gene, and as such the disease severity is determined by *SMN2* gene copy numbers. Although SMN protein plays diverse roles in RNA metabolism and is expressed ubiquitously throughout the body, deficiency in SMN protein affects primarily the motor system in SMA [3]. Recent studies using various animal models have greatly expanded our understanding of SMA at the cellular and molecular levels [4]. However, the cellular basis and pathogenesis of motor impairment in SMA remain unclear [5].

One proposed idea is that motor impairment in SMA may result from motoneuron loss and peripheral denervation. In a widely used SMA mouse model (SMNΔ7) that recapitulates many symptoms of human SMA, denervation (∼7–15%) is indeed identified in a few proximal muscles, such as the paraspinal and intercostal muscles when motoneuron loss is modest at the end stage [6], [7], [8]. However, no denervation is observed in a wide range of major limb muscles [6], [9]. Thus, it remains intriguing why the ambulatory function is impaired. In addressing the structural and functional integrity of neuromuscular junctions (NMJs), recent studies reported NMJ pathologies, such as neurofilament accumulation and immature endplate morphology, as well as a reduction in quantal release in SMNΔ7 mice [6], [8], [9], [10]. However, given the high safety factor at the NMJ [11], it is unclear whether the reduction of transmitter release would be severe enough to cause neuromuscular transmission failure and muscle weakness in the non-denervated muscle targets in SMNΔ7 mice. Thus, further functional analyses of the NMJ and muscle contraction in SMNΔ7 mice would resolve the role of NMJs in muscle weakness.

Besides the NMJ and muscles, motor behavior is also governed by neural circuits in the spinal cord, where motoneurons receive synaptic inputs from local interneurons, descending pathways and proprioceptive sensory neurons. The convergence of proper excitatory and inhibitory inputs onto motoneurons is required for motor control, reflexes and tonic firing of the motoneurons. Disruption of the cellular components and/or connectivity in this spinal circuitry has been implicated in the motoneuron disease [12], [13], [14]. However, little is known about the synaptic connectivity in the SMA spinal cord, and whether or not the loss of central synapses participates in the pathogenesis of SMA is an open question.

Given the emerging concept that many neurodegenerative diseases involve synaptopathy [15], it is crucial to study synapses at multiple levels of the spinal and neuromuscular circuitry in SMA. In the current study, we used SMNΔ7 mice to examine the synaptic circuitry controlling hindlimb muscles responsible for ambulatory function, which is compromised in both patients and mice [16], [17]. We showed that NMJs at the SMNΔ7 hindlimb, despite being structurally and functionally altered, were capable of eliciting muscle contraction upon nerve stimulation. In addition, we found that synapses onto spinal motoneurons in the lateral column of lumbar segments 3–5 were significantly reduced in mutant mice. The novel concept that SMA is a disease involving central, in addition to peripheral, synaptopathy may lead to new therapeutic approaches that work to enhance synapse formation and function in the spinal cord.

The preliminary results of this study have been presented (K.K.Y.L., M.Y.L., C.P.K. 2007 Society for neuroscience meeting abstract. #488.7; 2008 Society for neuroscience meeting abstract. #643.6).

## Results

### NMJs in SMNΔ7 hindlimb muscles are fully innervated throughout the disease progression

**Figure 1:**
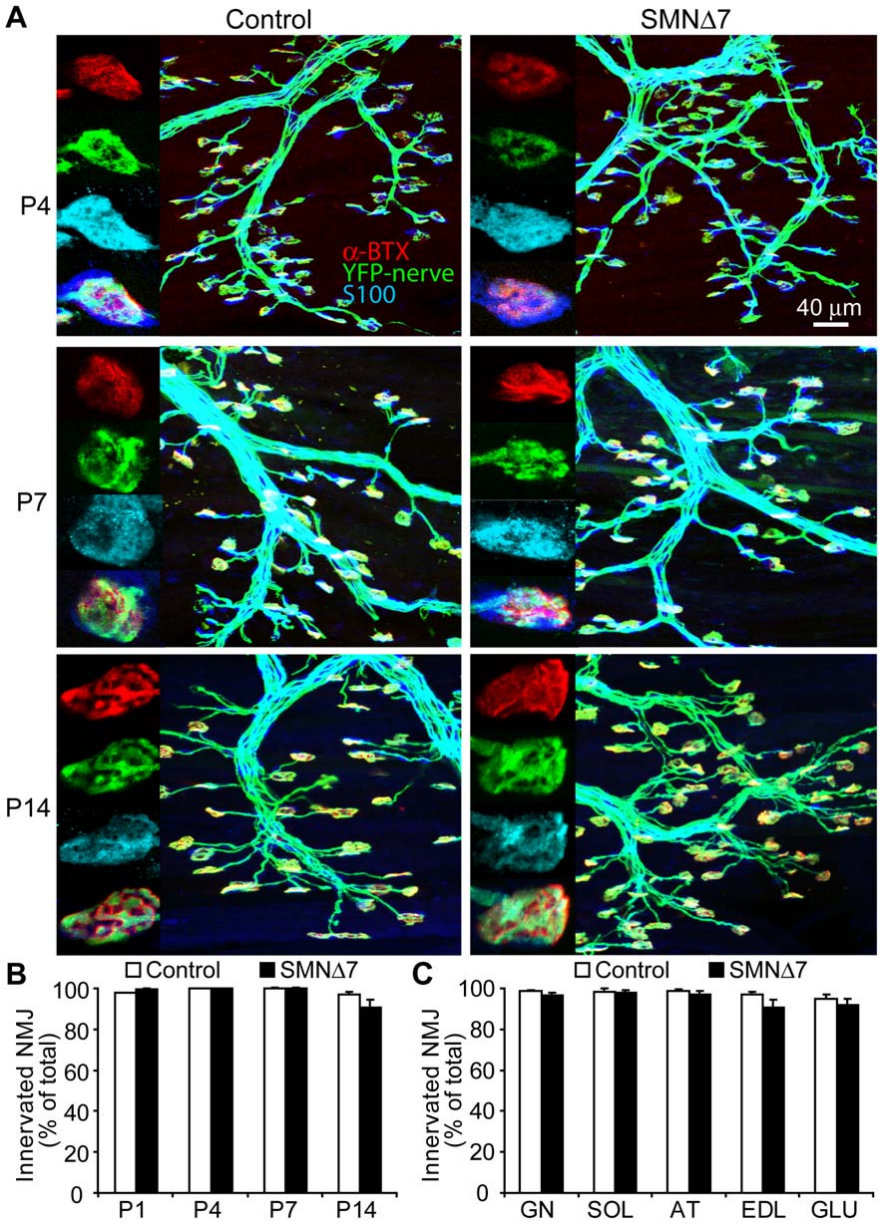
Fully innervated NMJs in hindlimb muscles of SMNΔ7 mice that express YFP in motoneurons. (**A**) Z-stacks of confocal micrographs showing typical NMJs in extensor digitorum longus (EDL) of the SMNΔ7 and control mice at P4, P7 and P14. Nerve terminals overexpressing YFP (in green), the postsynaptic acetylcholine receptor (AChR) clusters (in red) and the perisynaptic Schwann cells (PSCs; in blue). (**B**) Bar graph of the percentage of fully innervated NMJs over total number of endplates at EDL in control and SMNΔ7 mice at P1, P4, P7 and P14. (**C**) Histogram showing the percentage of fully innervated NMJs over total number of endplates at P14 hindlimb muscles (GN, gastrocnemius; SOL, soleus; AT, anterior tibialis; GLU, gluteus maximus). N>300 NMJs in 3–6 animals in each genotype. Error bars show s.e.m.

Prior to addressing whether a defect in neuromuscular function contributes to motor impairment, it is essential to examine the innervation pattern of NMJs in major hindlimb muscles in SMNΔ7 mice. To facilitate the observation of the nerve terminals, we crossed a mouse line that overexpresses yellow fluorescent protein (YFP) in motoneurons [18] with the SMNΔ7 mouse line. Similar to the original SMNΔ7 mouse line [19], the SMNΔ7/YFP mice displayed progressive muscle weakness with a life span of ∼14 days. We labeled perisynaptic Schwann cells (PSCs) with anti-S100 antibody and postsynaptic acetylcholine receptors (AChRs) with α-bungarotoxin to examine the tripartite arrangement of the NMJ [20]. Throughout disease progression (postnatal day 1–14), we found that nearly all NMJs in the extensor digitorum longus (EDL) muscle of SMNΔ7 mice, as in non-SMA control littermates (i.e. littermates that carry homozygous/heterozygous mouse *Smn* alleles), were fully innervated (Fig. 1A and 1B). Even at the end-stage, we observed only an insignificant number of NMJs that were denervated (control vs. SMNΔ7: total denervation, ∼1% vs. ∼5%; partial denervation, ∼2% vs. ∼4%) in the EDL. Similarly, we also observed that nearly all SMNΔ7 NMJs at the end-stage were fully innervated in a wide range of hindlimb muscles types, including predominantly fast muscle type (EDL, tibialis anterior), slow muscle type (soleus), mixed type (gastrocnemius), and the most proximal limb muscle, the gluteus maximus (Fig. 1C). Moreover, PSCs were co-localized with over 99% of nerve-muscle contacts in both fast and slow muscle types examined (Fig. 1A and Fig. S1). We did not observe PSC sprouting, a feature often seen following denervation [21]. The innervation pattern was further confirmed with immunocytochemistry using antibodies against neurofilament and synaptophysin (data not shown). As previously reported in various SMA mouse models [6], [8], [9], [10], [22], [23], we also observed neurofilament accumulation in the majority of SMNΔ7 NMJs (Fig. S2). In short, our findings suggest that nearly all NMJs in SMNΔ7 hindlimb muscles are fully innervated.

### Reduced neuromuscular transmission in SMNΔ7 hindlimb muscles

**Figure 2:**
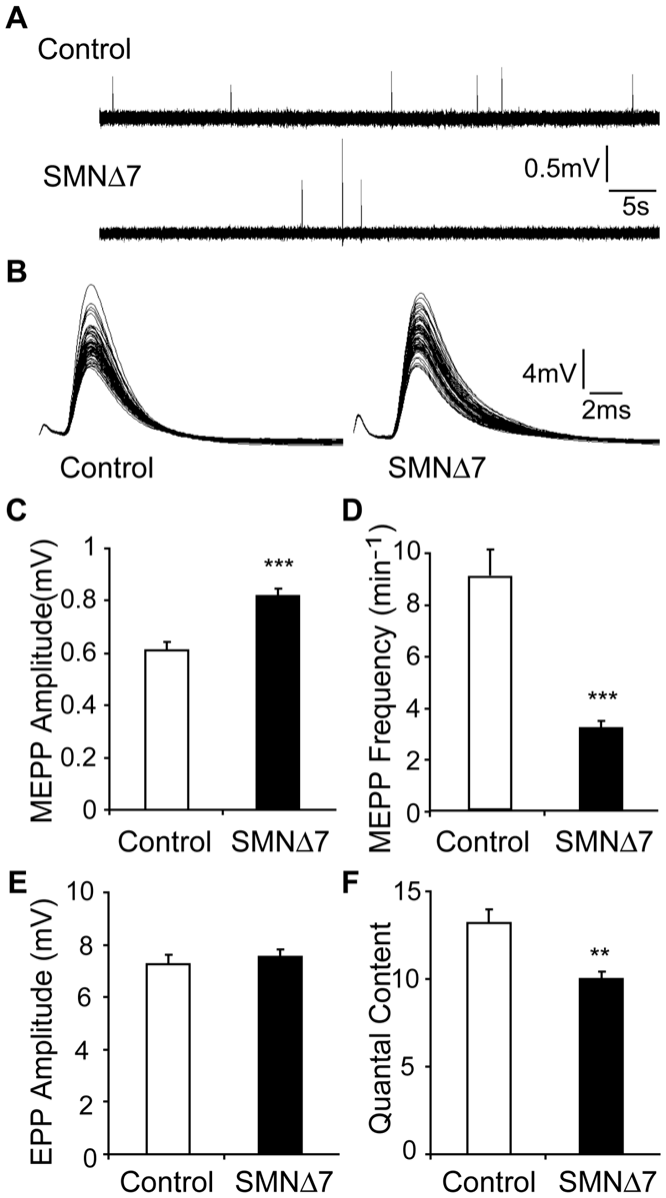
SMNΔ7 NMJs display a decrease in synaptic efficacy. Intracellular recording was performed in EDL muscle at P12–P14. (**A**) Sample recordings of MEPP from control (top) and SMNΔ7 (bottom) mice. (**B**) Sample recordings of EPP from control (left) and SMNΔ7 (right) mice. (**C–F**) Quantifications of the amplitude and frequency of MEPPs (MEPP amplitude: control 0.61±0.03 mV vs. SMNΔ7 0.82±0.03 mV, *p*<0.0001; MEPP frequency: control 8.97±0.99 min^−1^ vs. SMNΔ7 3.16±0.26 min^−1^, *p*<0.0001;), EPP amplitude (control 7.27±0.34 mV vs. SMNΔ7 7.53±0.31 mV, *p* = 0.58) and quantal content (control 13.20±0.77 vs. SMNΔ7 9.97±0.45, *p* = 0.007). There is an increase in MEPP amplitude, a decrease in MEPP frequency and a reduction in quantal content in SMNΔ7 mice (n = 100–130 NMJs from 7 control and 10 SMNΔ7 mice; ***p<0.0001, ** *p* = 0.007). Error bars show s.e.m.

To examine neuromuscular transmission in SMNΔ7 mice, we performed intracellular recording in EDL muscles at the disease end-stage (P12–14). Muscle contraction was prevented by pre-incubation of μ-conotoxin, which blocks voltage-gated sodium channels in muscle [24]. Spontaneous miniature endplate potentials (MEPPs) and evoked endplate potentials (EPPs) could be recorded from almost all junctions in both control and SMNΔ7 mice. SMNΔ7 NMJs showed a significantly reduced MEPP frequency and increased MEPP amplitude (Fig. 2A, 2C and 2D). The small increase in MEPP amplitude is probably due to the higher input resistance of the smaller SMNΔ7 muscle fibers. However, we found that the EPP amplitude was similar between control and SMNΔ7 NMJs (Fig. 2B and 2E). Correspondingly, quantal content, calculated by dividing the amplitude of EPP over MEPP [25], was reduced by about 25% in SMNΔ7 NMJs (Fig. 2F).

**Figure 3:**
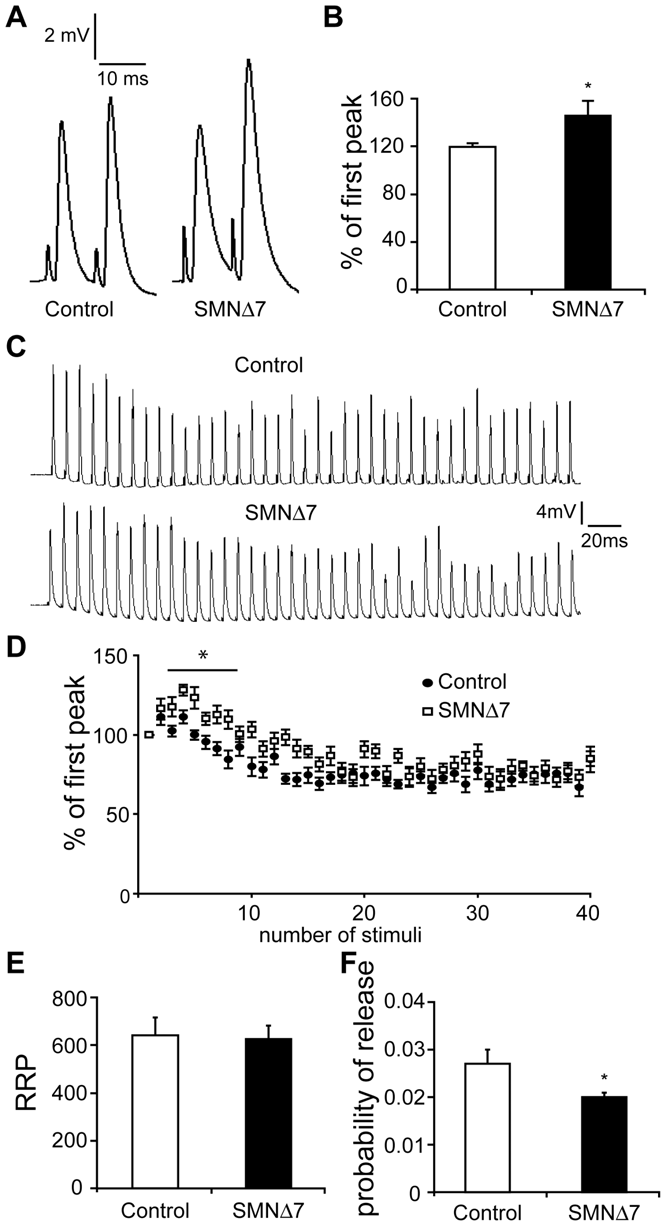
SMNΔ7 NMJs exhibit lower vesicle release probability. Intracellular recording was performed in EDL muscle at P12–P14. (**A**) Example of paired-pulse response from control (left) and SMNΔ7 (right) NMJs. (**B**) SMNΔ7 NMJs showed stronger paired-pulse facilitation indicating a lower vesicle release probability (control 119.4±3.54% vs. SMNΔ7 145.9±12.5%; *p* = 0.02; n = 14 NMJs from 2 control mice and 9 NMJs from 2 SMNΔ7 mice; * *p* = 0.02). (**C**) Sample traces of high frequency (50 Hz) stimulation responses from control NMJs (top) and SMNΔ7 NMJs (bottom). (**D**) Synaptic responses normalized to the first EPP. No transmission failure was observed in both control and SMNΔ7 mice. (**E**) RRP size is not different between control and SMNΔ7 NMJs (control 640.2±77.1 vs. SMNΔ7 625.4±54.8, *p* = 0.9). (**F**) Probability of release is significantly lower at SMNΔ7 NMJs (control 0.027±0.003 vs. SMNΔ7 0.02±0.001, *p* = 0.03; n = 15 NMJs from 5 control mice and 14 NMJs from 6 SMNΔ7 mice). Error bars show s.e.m.

The reduced quantal content may have resulted from a decrease in vesicle release probability, a smaller readily releasable pool (RRP) or both. We thus subjected the NMJs to paired-pulse and 50 Hz stimulations to examine whether SMNΔ7 NMJs show differences in release probability and RRP size. There was an increase in paired-pulse facilitation in SMNΔ7 NMJs (Figs. 3A and 3B), indicating a lower vesicle release probability [26]. Upon 50 Hz stimulation, similar to control NMJs, SMNΔ7 NMJs were able to sustain repetitive stimulations without any failure (Figs. 3C and 3D). Although SMNΔ7 NMJs exhibited more facilitated responses at the beginning of the stimulus train, they depressed to a similar level as control NMJs. In addition, we calculated the RRP size using the method by Elmqvist and Quastel [27], and found a similar RRP size in control and SMNΔ7 NMJs (Fig. 3E). The probability of release, calculated by dividing the quantal content of the first EPP by RRP size [28], however, is significantly lower by 26% at SMNΔ7 NMJs (Fig. 3F). These data suggest the reduced quantal content in SMNΔ7 hindlimb NMJs is likely due to a decrease in vesicle release probability, rather than a reduction of RRP size.

### NMJs in SMNΔ7 hindlimb muscles are capable of eliciting muscle contraction

**Figure 4:**
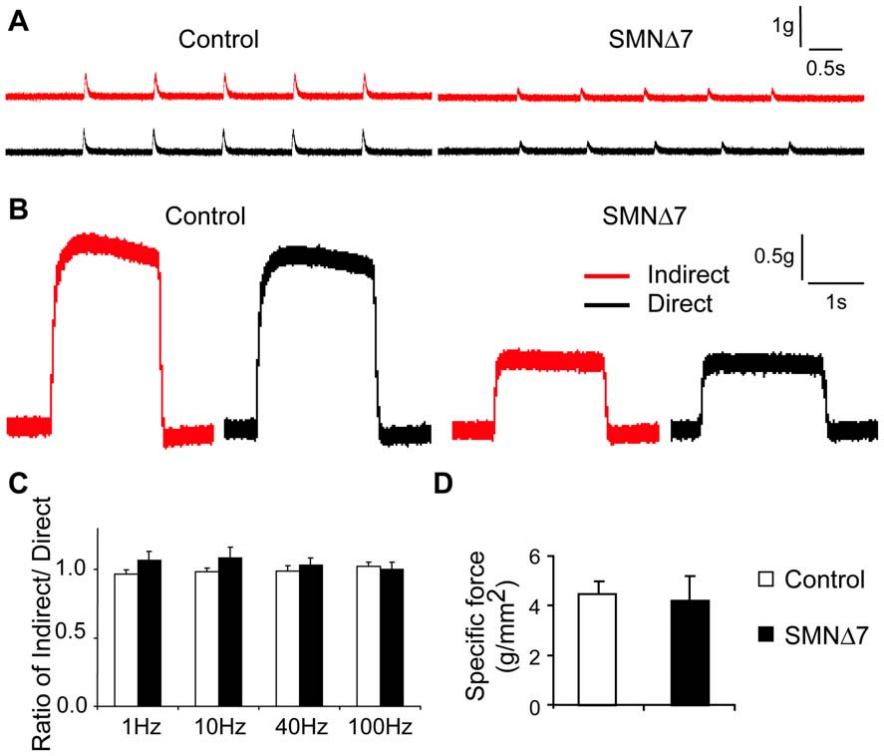
SMNΔ7 NMJs can elicit muscle contraction at disease end-stage. (**A**) Sample traces of muscle twitch tension measurements at 1 Hz. (**B**) Sample traces of muscle tetanic force measurements at 100 Hz. In both control (left) and SMNΔ7 (right) EDL muscles, indirect nerve (red) and direct muscle (black) stimulation elicited similar amounts of muscle force. (**C**) Quantification of the ratio of indirect over direct stimulation evoked muscle contraction at 1, 10, 40, 100 Hz. The ratio was close to one at all frequencies examined, indicating overall NMJ function is not compromised in SMNΔ7 mice (control vs. SMNΔ7: 1 Hz, 0.97±0.03 vs. 1.07±0.07; 10 Hz, 0.98±0.02 vs. 1.09±0.08; 40 Hz, 0.99±0.04 vs. 1.03±0.05; 100 Hz, 1.02±0.03 vs. 1.00±0.05; n = 9 control muscles and 9 SMNΔ7 muscles). (**D**) Specific force is not significantly different in SMNΔ7 and control mice (control 4.48±0.54 g/mm^2^ vs. SMNΔ7 4.23±0.99 g/mm^2^, *p* = 0.84; n = 7 control muscles and 8 SMNΔ7 muscles). Error bars show s.e.m.

To address whether the reduced synaptic efficacy at the NMJ would lead to failures in muscle contraction, we measured muscle twitch tension in response to the indirect (nerve) and direct (muscle) stimulation at various frequencies (1, 10, 40, 100 Hz) in EDL muscles at the disease end-stage (Fig. 4). If the amount of neurotransmitter release is sufficient to trigger muscle contraction, the force generated through nerve and muscle stimulation is expected to be the same and the ratio of indirect/direct stimulation should be close to one. However, if neuromuscular transmission failures occur, the ratio would be less than one. As expected, we found that in control muscle the single twitch tension (1 Hz, Fig. 4A) as well as the tetanic force (100 Hz, Fig. 4B) generated by nerve stimulation were similar to those by muscle stimulation. We found that this was also the case for SMNΔ7 muscle (Fig. 4A and 4B). As quantified in Fig. 4C, the ratio of indirect over direct stimulation evoked muscle tension was close to one at all frequencies examined in both control and SMNΔ7 mice. These results suggest that the overall neuromuscular transmission is not compromised in SMNΔ7 hindlimb muscles, despite the reduction by approximately 25% in quantal content.

We did find that SMNΔ7 muscles produced weaker tension (Fig. 4A and 4B), and had smaller muscle size compared to control muscles (cross-section area: Control vs. SMNΔ7, 0.46±0.02 vs. 0.23±0.02 mm^2^). To examine whether the reduced force production can be attributed to the size difference, the specific force was calculated by normalizing the maximum muscle tension (by direct muscle stimulation at 100 Hz) to the muscle cross-section area. We found that the specific force was not significantly different between control and SMNΔ7 mice (Fig. 4D). These data suggest that the basic muscle contractile ability may not be significantly affected and the reduced muscle force was likely contributed by the smaller muscle size in SMNΔ7 mice.

### Loss of central synapses onto L3–5 lateral spinal motoneurons in SMNΔ7 mice

**Figure 5:**
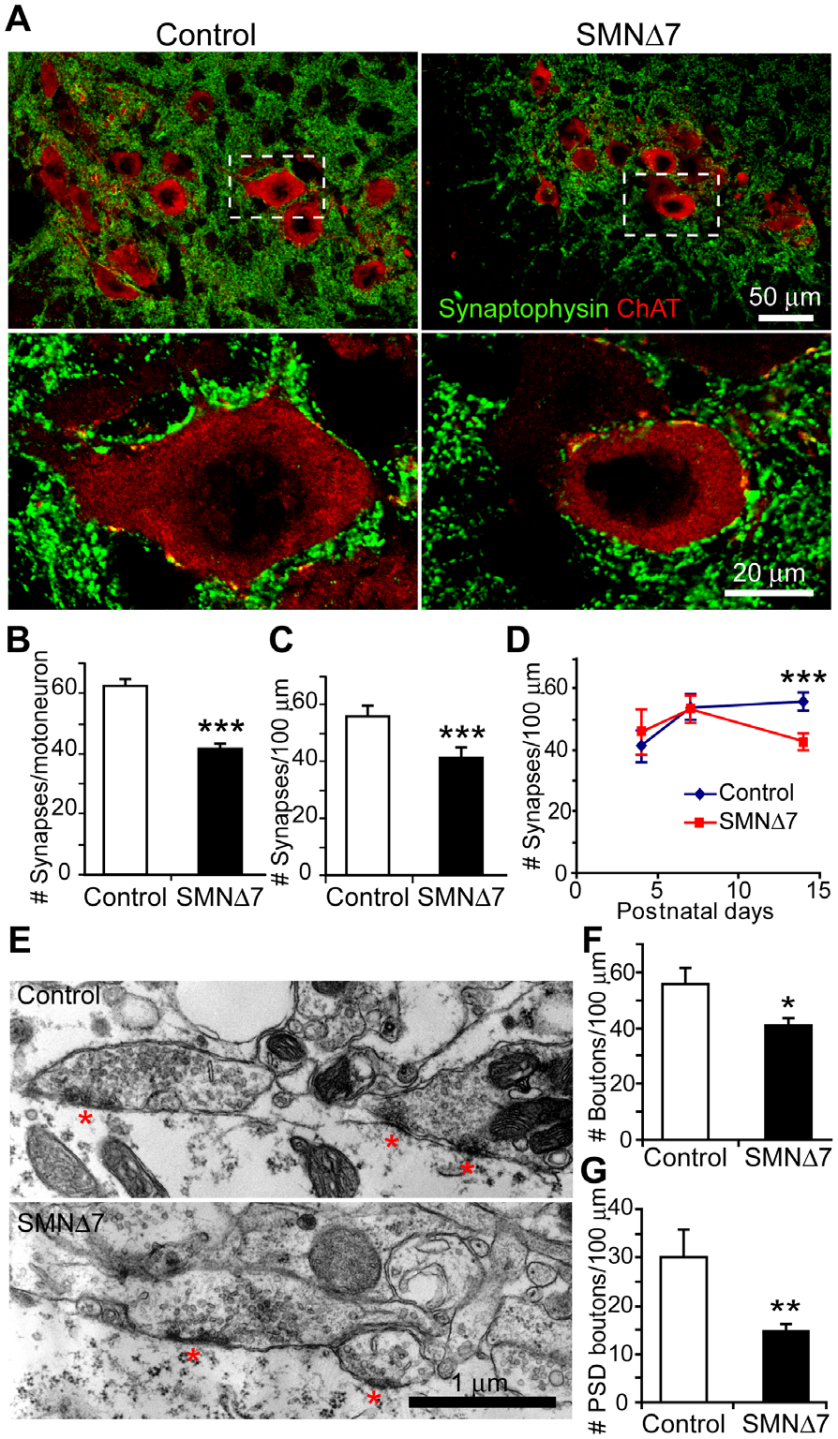
Synapses onto L3–5 lateral motoneurons are reduced in SMNΔ7 mice at the end-stage. (**A**) Confocal micrographs showing the synapses (labeled with anti-synaptophysin, green) and motoneurons (labeled with anti-ChAT, red) in the ventral horn of the spinal cords of control and SMNΔ7 mice. (**B**) Bar graph showing the mean number of synapses on control (64.3±3.6, n = 180 motoneurons) and SMNΔ7 motoneurons (46.1±3.7, n = 163 motoneurons) (N = 10 animals for each genotype; *p*<0.0001). (**C**) Bar graph representing the mean number of synapses per 100 µm motoneuron perimeter on control (56.4±3.2) and SMNΔ7 (44.4±2.7) motoneurons (*p*<0.0001). (**D**) Bar graph showing synaptic density on motoneurons at P4 (control, 41.4±5.2, n = 64 neurons; SMNΔ7, 45.8±7.4, n = 46 neurons in 3 pairs of animals), P7 (control, 53.9±4.1, n = 48 neurons in 3 animals; SMNΔ7, 53.2±4.6, n = 83 neurons in 5 animals) and P14 (see **B**). (**E**) Electron micrograph showing axosomatic synapses on L3–5 lateral motoneurons in control and SMNΔ7 mice. (**F**) Bar graphs showing the density of presynaptic terminals on motoneurons (control vs. SMNΔ7: 55.8±5.8 vs. 40.8±6.0 boutons per 100 µm perimeter, *p = 0.024; Control: n = 8 neurons in 4 animals; SMNΔ7: n = 11 neurons in 4 animals). (**G**) Bar graphs showing the density of synaptic boutons juxtaposed to postsynaptic density (red asterisks in **E**) (control vs. SMNΔ7: 30.2±5.7 vs. 14.7±2.0 boutons per 100 µm motoneuron perimeter, **p = 0.009). Error bars show s.e.m.

In addition to the anatomical and functional connectivity of the NMJ, innervation of motoneurons in the spinal cord is also necessary to complete the motor circuitry involved in motor control and reflexes. To explore the possible deficiency in central synapses contributing to SMA pathology, the number of synapses on lumbar (L3–5) spinal motoneurons in SMNΔ7 mice at the end-stage (P12–14) were examined (Fig. 5). We examined only the motoneurons in the lateral column of these segments, which are known to innervate hindlimb muscles [29]. Synapses were fluorescently labeled with anti-synaptophysin antibody and identified as punctae, and α-motoneurons were identified by size (>300 µm^2^) [30], location and the immunoreactivity of anti-choline acetyltransferase (ChAT) (Fig. 5A). Quantification of synapses apposed to the soma and proximal dendrites of the ChAT-positive motoneurons with nuclei in SMNΔ7 mice as compared to control revealed an average ∼28% reduction in the number of synapses per motoneuron (Fig. 5B). In order to minimize the effect of potential size variance of sampled motoneurons, we calculated the synaptic density by normalizing the synapse number to unit length of motoneuron perimeter. This revealed a significant ∼21% reduction in SMNΔ7 mice compared to control mice (Fig. 5C). To exclude the possibility that the synapse count included synapses on dying cells, the structural integrity of the SMNΔ7 motoneurons was examined with hematoxylin and eosin (H&E) staining subsequent to confocal imaging. Neither necrotic changes (indicated by eosinophilic cytoplasm) nor apoptotic bodies were associated with the SMNΔ7 motoneurons sampled (Fig. S3).

To address whether this reduction in synapses is attributed to a defect in synaptic formation or maintenance, we examined the synaptic density on L3–5 spinal motoneurons at earlier developmental stages, P4–P7, when perinatal synaptogenesis continues [31]. We found that, in SMNΔ7 mice, the synaptic density at both P4 and P7 were similar to that in control mice (Fig. 5D). However, unlike control motoneurons, SMNΔ7 motoneurons displayed synapse loss between P7 and P14. Thus, SMNΔ7 motoneurons in L3–5 lateral spinal columns are capable of forming new synapses during early postnatal development, but synapses are not maintained as the disease progresses.

We also examined synapse loss on SMNΔ7 L3–5 lateral motoneurons at the electron microscopic level (Fig. 5E). We found a ∼26% reduction in the number of presynaptic terminals onto motoneurons (identified by size and location) when comparing SMNΔ7 to control (Fig. 5F). This is consistent with the results we obtained using confocal microscopy as described above. When we used a more stringent morphological criterion to define synapses by quantifying only the presynaptic boutons at juxtaposition to the electron-dense postsynaptic density (PSD, red asterisks in Fig. 5E), a more extensive loss of synapses (∼50%; Fig. 5G) in SMNΔ7 was observed. Furthermore, although we found no significant difference in the presynaptic bouton size between control and SMNΔ7, we did observe a ∼34% reduction in the size of postsynaptic density relative to the presynaptic bouton size (Fig. S4).

### Selective reduction of glutamatergic synapses on L3–5 lateral spinal motoneurons in SMNΔ7 mice

**Figure 6:**
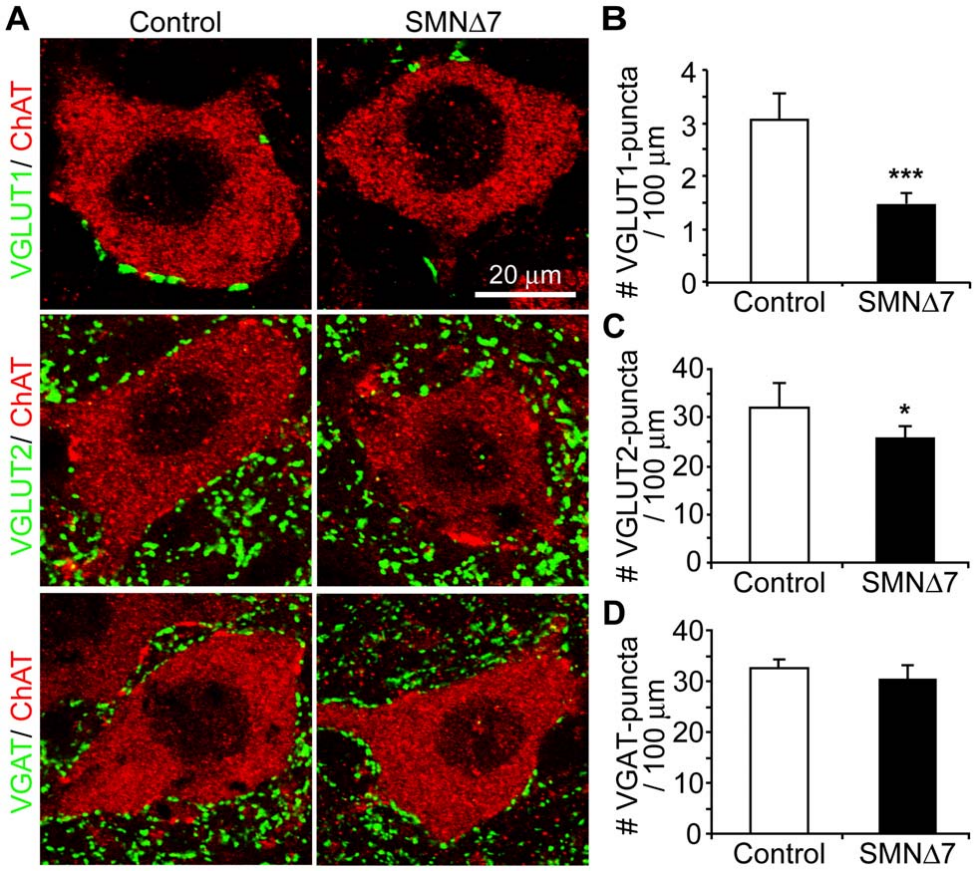
Glutamatergic synapses onto L3–5 lateral motoneurons are reduced in SMNΔ7 mice at the end-stage. (**A**) Confocal images showing glutamatergic and GABAergic synapses in the ventral horn of the lumbar spinal cord in control and SMNΔ7 mice. Glutamatergic nerve terminals were labeled with anti-vesicular glutamate transporter (VGLUT)1 and 2 (green), GABAergic terminals with anti-vesicular GABA transporter (VGAT; green), and motoneurons with anti-ChAT (red). (**B**) Bar graph of the number of VGLUT1-positive synapses per 100 µm motoneuron perimeter on control and SMNΔ7 motoneurons (control 3.1±0.5 vs. SMNΔ7 1.5±0.4 boutons per 100 µm motoneuron membrane, n = 150 neurons in 5 control mice and n = 127 neurons in 5 SMNΔ7 mice; *p*<0.0001). (**C**) Bar graph showing the number of the VGLUT2-positive synapses on control and SMNΔ7 motoneurons (control, 32.1±4.1, n = 100 neurons; SMNΔ7, 27.0±2.5, n = 86 neurons, in 5 animals for each genotype; **p = 0.002). (**D**) Bar graph representing the number of the VGAT-positive synapses on motoneurons in control and SMNΔ7 mice (control, 32.6±1.8, n = 78 neurons; SMNΔ7, 30.9±3.1, n = 73 neurons, in 3 pairs of animals). Error bars show s.e.m.

To explore the possible sources of the synapse reduction, we examined different types of spinal synapses at the end-stage (P12–14) in SMNΔ7 mice. The number of glutamatergic excitatory synapses was examined using anti-vesicular glutamate transporters (VGLUT) 1 and 2 that label the majority of glutamatergic synapses in the spinal cord [32]. GABAergic inhibitory synapses were identified with anti-vesicular GABA transporters (VGAT) [33]. We found that there was an average ∼54% reduction in the number of VGLUT1 synapses onto the soma and proximal dendrites of L3–5 lateral spinal motoneurons in SMNΔ7 mice as compared to control littermates (Fig. 6A and 6B). In addition to VGLUT1, the number of VGLUT2-positive synapses also showed a slight but significant reduction of ∼14% onto L3–5 lateral spinal motoneurons in SMNΔ7 mice (Fig. 6A and 6C). No reduction of either VGLUT1 or VGLUT2 synapses was observed at P7 (Fig. S5). In contrast to glutamatergic synapses, there was no significant change in the number of GABAergic synapses onto SMNΔ7 motoneurons at the end stage (Fig. 6A and Fig. 6D). Taken together, the reduction in synapses onto SMNΔ7 motoneurons is likely contributed by the decreased glutamatergic excitatory synapses.

### Reduction of proprioceptive sensory neurons in SMNΔ7 mice

**Figure 7:**
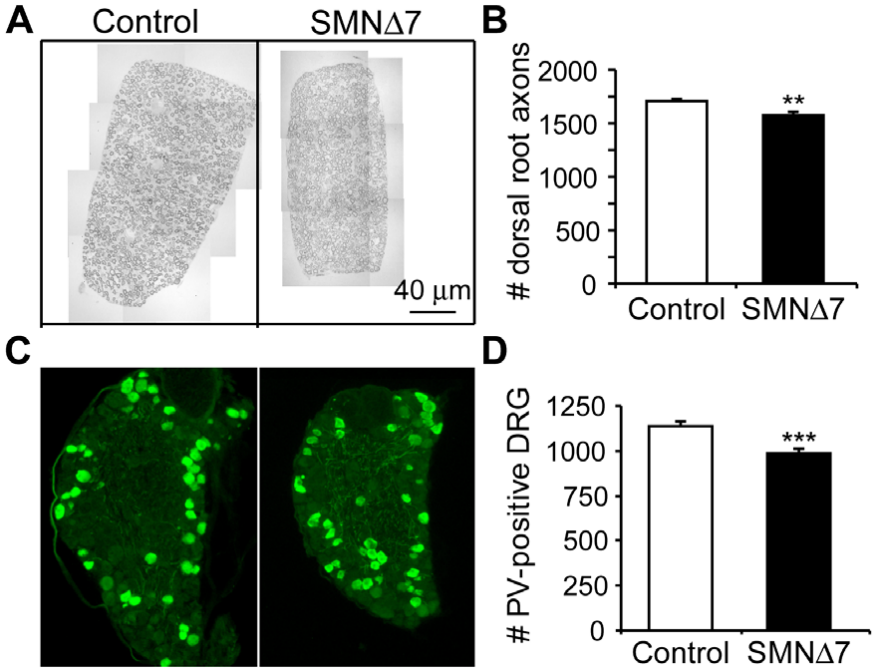
Proprioceptive sensory axons and neurons are reduced in SMNΔ7 mice. (**A**) Sample pictures showing the cross section of lumbar 4 (L4) dorsal root axons from control and SMNΔ7 mice at P14. (**B**) Bar graph showing the average number of myelinated dorsal root axons in control (1707±20, n = 6) and SMNΔ7 mice (1577±27, n = 7), *p = 0.0032. (**C**) Micrographs showing proprioceptive neurons immuno-labeled with anti-parvalbumin (PV) antibody (in green) in the L4 dorsal root ganglion (DRG) of control and SMNΔ7 mice. (**D**) Bar graph of the total number of PV-positive cells in L4 DRGs in control (1138±27, n = 6) and SMNΔ7 (985±29, n = 4) mice, *p*<0.0001. Error bars show s.e.m.

It is known that a vast majority of the VGLUT1-positive synapses abutting onto motoneurons primarily originate from proprioceptive sensory neurons [32]. To examine whether an alteration in proprioceptive input from sensory axons is involved in SMA pathology, the number of proprioceptive dorsal root ganglion (DRG) cells and their axons was examined in the lumbar spinal segment (L4) of SMNΔ7 mice at disease end-stage (P12–14). Fig. 7A shows the gross morphology of a dorsal root from control and SMNΔ7 mice. We found a slight but significant reduction (∼8%) in the average number of myelinated dorsal root axons in SMNΔ7 mice compared to that of control littermates (Fig. 7B). Using an antibody against parvalbumin (PV) as a marker for proprioceptive DRG cells [34] (Fig. 7C), we found a significant ∼13% reduction in the number of proprioceptive DRG cells in L4 spinal segment of SMNΔ7 mice (Fig. 7D).

### Increased association of microglia and motoneurons in the spinal cord of SMNΔ7 mice

**Figure 8:**
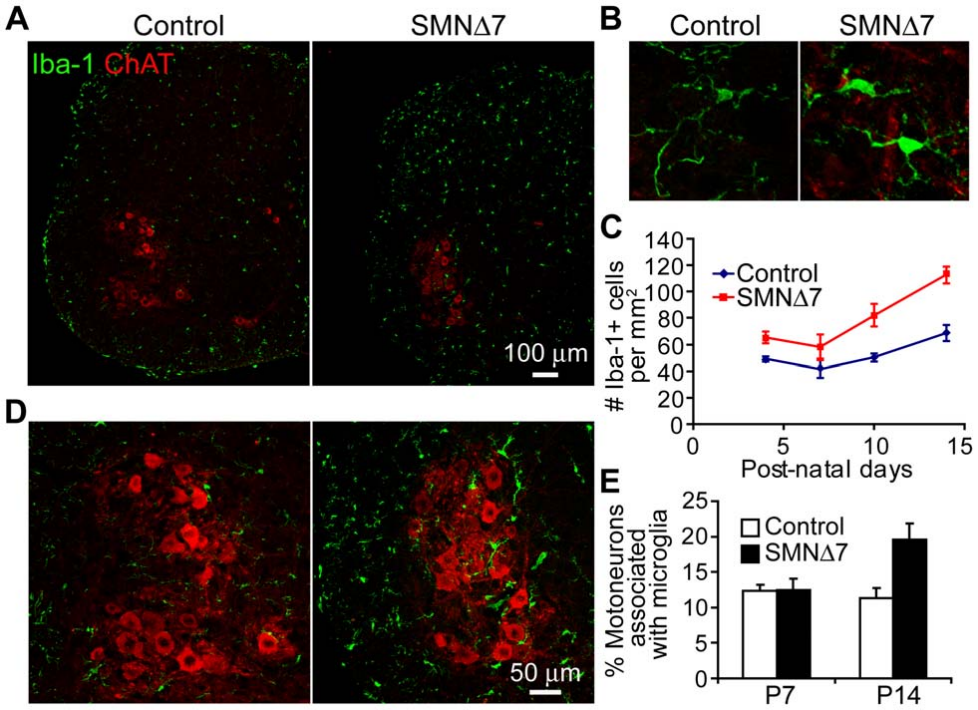
Microglia associate with lateral motoneurons in SMNΔ7 lumbar (L3–5) spinal cord. (**A**) Confocal images showing microglia (labeled with anti-Iba-1, green) and motoneurons (labeled with anti-ChAT, red) in the hemi-section of the lumbar spinal cord of control and SMNΔ7 mice at P14. (**B**) Magnified views of representative microglia in control and SMNΔ7 lumbar spinal cord at P14. (**C**) Bar graphs showing the density of Iba-1-positive cells in the lumbar spinal cord in control and SMNΔ7 mice during development. Control vs. SMNΔ7 (N = 4 for each genotype): P4, 49.0±2.1 vs. 65.5±4.5 (*p* = 0.048) ; P7, 41.7±6.6 vs. 58.3±9.0 (*p* = 0.180); P10, 50.1±3.2 vs. 82.1±8.7 (*p* = 0.029); P14, 68.4±6.1 vs. 112.7±6.2 (*p* = 0.002). (**D**) Confocal images showing microglia (green) and motoneurons (red) in the ventral horn of the lumbar spinal cord of control and SMNΔ7 mice. (**E**) Bar graphs showing the percentage of motoneurons associated with microglia at P7 (control 12.4±0.9% vs. SMNΔ7 12.5±16%, N = 4 for each genotype) and P14 (control 11.3±1.4% vs. SMNΔ7 19.6±2.2%, N = 6 for each genotype; *p* = 0.01). Error bars show s.e.m.

Microglia are the resident inflammatory cells in the central nervous system which could mediate synaptic turnover during development and in diseases with or without the context of neuroinflammation (see review [35]). To investigate the involvement of microglia in SMA, we used anti-Iba-1 antibody [36] to label microglia in the lumbar (L3–5) spinal cord of SMNΔ7 mice before and after synapse loss (i.e. P4–P14) (Fig. 8). During normal development, the density of microglia increased perinatally in the central nervous system (Fig. 8C; also see review [37]). In SMNΔ7 mice, we found a ∼34% increase in the density of microglia at P4 and a more profound increase (∼65%) at P14 when compared to control mice (Fig. 8A and 8C). In addition, microglia in SMNΔ7 mice appeared to be activated as demonstrated by their larger cell body with intense Iba-1 immunoreactivity, and shorter, thicker processes [35], [36] (Fig. 8B). Furthermore, we observed a robust increase in its physical association with motoneurons in SMNΔ7 lumbar spinal cord at P14 compared to P7 (Fig. 8D and 8E), which would suggest a possible involvement of microglia-mediated synaptic stripping [38].

## Discussion

The present study investigated the possible involvement of peripheral and central synaptopathy of the motor circuitry in the SMNΔ7 mouse model of SMA. We found that nearly all NMJs in the SMNΔ7 hindlimb muscles were innervated and capable of neurotransmitter release even at the end-stage when motor impairment was evident. Despite a modest reduction in synaptic efficacy in SMNΔ7 NMJs at the end-stage, such a reduction was not sufficient to cause failure in muscle contraction. In the spinal cord, we found a significant reduction in glutamatergic central synapses abutting on the soma and proximal dendrites of SMNΔ7 motoneurons, which was, in part, likely contributed by a loss of proprioceptive sensory neurons. Furthermore, we showed an increase in microglia number and association with motoneurons in SMNΔ7 mice. Our findings provide new insights into the disease pathology of SMA and may lead to the development of novel therapeutic strategies that include central synaptic targets.

### Synaptic defects at the NMJ do not result in transmission failure in SMNΔ7 hindlimb muscles

Our morphological findings using both genetically expressed YFP and immuno-histological staining are consistent with recent studies showing the majority of NMJs in hindlimb muscles are fully innervated in SMNΔ7 mice [6], [9]. One potential concern is that the fully innervated NMJs we observed may have resulted from a delay in synapse elimination, which occurs during the first two weeks after birth [39]. However, this seems unlikely as synapse elimination at NMJs [6], [9] and the motor unit size are not altered in hindlimb muscles of SMNΔ7 mice [Lee Y., Mikesh M., Rimer M., Thompson W. 2007 Society for Neuroscience meeting abstract. #490.9].

The 25% reduction in quantal release we found in the EDL muscle is consistent with the 50% decrease observed in another hindlimb muscle, the tibialis anterior [6], as well as in proximal muscles, such as transversus abdominis and levator auris longus muscles [10]. However, since a faithful neuromuscular transmission is so critical for the survival of an organism, motor nerve terminals typically release 2 to 20 times more neurotransmitters than necessary in mice[11]. The functional consequence of the 25 to 50% reduction in synaptic efficacy might thus be negligible as our twitch tension measurement experiments demonstrated. This is in agreement with a recent study showing that muscle fibers could fire normal action potentials in response to nerve stimulation despite a 50% reduction in quantal release in SMNΔ7 mice [10]. Thus, it seems unlikely that the motor impairment in the hindlimb muscles of SMNΔ7 mice is simply caused by muscle denervation or neuromuscular transmission failure. Our twitch tension findings suggest that the ambulatory difficulties might be in part attributed to the reduced muscle force production as a result of the smaller muscle size and possible abnormal muscle development [Lee Y., Mikesh M., Rimer M., Thompson W. 2008 Society for Neuroscience meeting abstract. #752.3] in SMNΔ7 mice.

Although denervation is not observed in hindlimb muscles, it is important to note that denervation has been found in several proximal muscles, and would likely contribute to disease phenotypes seen in SMA patients and mouse models [6], [7], [8], [17], [23]. For example, the intercostal muscles are denervated in the severe SMA mouse model (*Smn* ^−/−^;*SMN2* ^+/+^), while the diaphragm muscle is relatively spared [7]. The denervation in the intercostal muscles may explain the clinical observations that breathing is almost entirely diaphragmatic in type I SMA patients [40]. The reason behind the selective degeneration of motor nerve terminals in different muscles remains elusive and why and how muscles show different vulnerability to denervation in SMA would require further investigation.

### Reduction in central synapses in the spinal cord of SMNΔ7 mice

A loss of central synapses is not uncommon in neurodegenerative diseases [41], [42]. Concordant evidence of synapse loss was observed in the ventral horn of autopsied spinal cords from amyotrophic lateral sclerosis (ALS) patients [43], [44], [45] and a mouse model of ALS [14], [46]. In studies of autopsied spinal cord tissues from the SMA Type I patients, a reduction in synaptophysin-immunoreactivity has also been reported in the ventral horn of the spinal cord [47], [48]. Although the authors concluded that the synaptophysin-immunoreactivity was preserved in most surviving motoneurons, no quantitative data was provided in the studies. Using multiple imaging approaches, we have shown in SMNΔ7 mice a significant reduction of central synapses, particularly the proprioceptive inputs, on the lateral motoneurons in lumbar spinal segments L3–5, which innervate the distal hindlimb muscles [29]. It is noted that a similar loss in proprioceptive inputs in L1 spinal segments of SMNΔ7 mice has also been reported in an abstract recently by Mentis et al. (2008, Society for Neuroscience meeting abstract. #643.22). Interestingly, the reduction in proprioceptive inputs is not only observed in SMNΔ7 mice, but also in a new mouse model of SMA [49]. Together, these findings raise the possibility that central synapse loss plays an important role in the pathogenesis of SMA.

Our data showed that the central synapse loss occurs at a stage when motoneuron loss is evident in the spinal cord of SMNΔ7 mice [19]. This raises the concern that synapse loss might be scored on dying motoneurons. To exclude this possibility, we performed H&E staining subsequent to confocal imaging of motoneurons to confirm that synapse loss indeed occurs on surviving SMNΔ7 motoneurons devoid of degenerative features (Fig. S3). However, the possibility that synapse loss results from secondary events in response to neurodegeneration, such as gliosis, cannot be totally ruled out. In fact, we did observe an increase in microglia population and their association with motoneurons in SMNΔ7 lumbar spinal segments. This is in line with the idea that microglia may contribute to the synapse loss through a process called synaptic stripping – a process in which microglia physically remove synaptic inputs from CNS neurons in response to insults in both central [50] and peripheral nervous systems [38]. However, what triggers the activation of microglia in SMNΔ7 mice would require further investigation.

Our findings of the reduction of VGLUT1-terminals associated with the reduction in L4 proprioceptive sensory neurons/axons suggest that sensory neurons and their afferents are affected by a deficiency in SMN protein. Indeed, there is evidence that sensory axons degenerate in SMA Type I patients [51], [52], [53]. Although signs of degenerating axons in the dorsal root of the SMNΔ7 mice were not observed, it is possible that degeneration only occurs at the distal collaterals of the dorsal root within the spinal cord. In addition, the reduction in synapses could also be attributed to the retarded growth of sensory axon terminals as implicated in an *in vitro* study of a more severe type of SMA mice (*Smn* ^−/−^;*SMN2* ^+/+^) [54]. However, we observed a similar density of VGLUT1 synapses on L3–5 lateral motoneurons in SMNΔ7 mice compared to that in control mice at P7 (Fig. S5). This suggests that Ia afferents are able to grow and find their targets in SMNΔ7 mice. It has been estimated that one single Ia afferent can sprout collaterals and form over 2700 synapses with up to 300 motoneurons in a motoneuron pool that innervates the medial gastrocnemius [55]. Therefore, even a relatively modest (∼13%) loss of proprioceptive neurons, as we observed in SMNΔ7 mice, could have a profound impact on the loss of synapses and function in a large number of spinal motoneurons.

The current study, like others that study central synapse loss in injured and diseased nervous systems [12], [14], [46], [56], [57], [58], [59], focuses only on soma and proximal dendrites. We have not addressed the possibility that synapses on soma vs. distal dendrites may display differential vulnerability. For example, selective loss of VGLUT1 synapse is observed on soma and proximal dendrites but not on distal dendrites after axotomy [60]. Even though synapses on the soma and proximal dendrites represent only a small proportion of all synapses abutting onto motoneurons, synapses on the somatodendritic membrane strongly influence the excitability of the motoneurons [61]. Therefore, the loss of somatodendritic synapses we observed in SMNΔ7 mice may have functional implications in motoneuron activity as discussed below.

### Potential functional implications of central synapse loss in SMA

Imbalanced neuronal activity has been associated with glutamate excitotoxicity in neurodegenerative diseases such as ALS [14], [62]. The reduction of excitatory synapses may potentially be neuroprotective, but could also reduce motoneuron activity that may impair motor control. In particular, we showed that in SMNΔ7 mice, the most drastic synapse reduction was found in the sensory-motor connection between proprioceptive sensory neurons and motoneurons. A permanent loss of VGLUT1 synapses in the proximal somatodendritic region of the motoneurons impairs stretch reflex response after peripheral nerve injury [60]. This might explain the abnormality in the sensory reflexes demonstrated by the abnormal gait, hindlimb splay, and the lack of grasp response in SMNΔ7 mice [16]. In addition, our finding is in line with reports that show the monosynaptic stretch reflex (H-reflex) is either decreased or absent in SMA patients [63], [64], [65].

The connectivity and functionality of both peripheral and central synapses are important for transmitting motor commands from the motor cortex. As discussed above, muscle weakness in hindlimb muscle is not a direct consequence of neurotransmission failure, but is likely contributed in part by a reduction in muscle size in SMNΔ7 mice. In addition, the lack of excitatory signals or imbalanced excitatory/inhibitory inputs onto motoneurons, as suggested in this study, may imply reduced excitation of α-motoneurons in SMA. Indeed, Mentis et al. (2008) have recently found that SMNΔ7 motoneurons show less synaptic response upon stimulation of dorsal root afferents and descending fibers from the thoracic spinal cord [Mentis G.Z., Sumner C.J., O'Donovan M.J. 2008 Society for Neuroscience meeting abstract. #643.22]. A recent study using multi-electrode recording of motoneurons on the spinal cord slices from another SMA mouse model (*Smn* ^−/−^; *SMN2* ^+/+^) also revealed reduced motoneuron activity [66]. Furthermore, defective synaptic inputs onto motoneurons have been shown to disrupt motor circuit activity in a *Drosophila* model of SMA [Imlach W., Savner E., Choi B., McCabe B.D. 2010. 14^th^ Annual International Spinal Muscular Atrophy Research Group Meeting hosted by Families of SMA]. Therefore, central synaptic defects could potentially affect the motoneuron activity and the motor circuitry, which may explain the deficit in motor coordination and reflex in SMA. It will be interesting to investigate whether or not functional changes in central synapses precede synaptic loss to account for early (∼P2) righting defects in SMNΔ7 mice [16]. Establishing central, in addition to peripheral, synaptopathy in the mouse model of SMA provides new insights into understanding SMA pathogenesis and designing treatment strategies.

## Materials and Methods

### Ethics Statement

The experimental procedures in this study were conducted in compliance with the US National Institutes of Health laboratory animal care guidelines. The protocol was approved by the Institutional Animal Care and Use Committee of the University of Southern California (protocol # 11136). All efforts were made to minimize the suffering of animals.

### Animals

Transgenic mice expressing SMA-like phenotypes were generated from breeder pairs obtained from Jacksons Laboratory (#5025; FVB.Cg-Tg(SMN2*delta7)4299Ahmb Tg(SMN2)89Ahmb Smn1^tm1Msd^). YFP-SMNΔ7 mice were generated by cross-breeding the aforementioned SMNΔ7 mice with a mouse-line expressing YFP driven by the Thy-1 promoter [18]. Genotypes of transgenic mice were determined as described before [18], [19]. Transgenic mice carrying either homozygous/heterozygous mouse *Smn* alleles were used as littermate controls.

### Immunohistochemistry of NMJs

Mice of the desirable genotype and age were anaesthetized by intraperitoneal injection of Nembutal (sodium pentobarbital; 50 mg/kg) or ketamine/ xylazine (100 mg/kg ketamine/ 10 mg xylazine) and transcardially perfused with Ringer's solution and then 4% paraformaldehyde. Whole muscles (gastrocnemius, soleus, tibialis anterior, extensor digitorum longus, gluteus maximus) were teased into layers of 5–10 fibers thick to facilitate penetration of antibodies that include: anti-synaptophysin antibody (Chemicon, 1∶400) for presynaptic nerve terminals, anti-S100 antibody (Dako, 1∶200) for Schwann cells and anti-neurofilament antibody (SMI 312, Covance, 1∶1000;). Acetylcholine receptors (AChRs) were labeled by Alexa Fluor 594-conjugated α-bungarotoxin (Invitrogen). Fluorescently labeled NMJs were observed with epifluorescence or confocal microscopes.

### Electrophysiology

The extensor digitorum longus (EDL) muscle from P12–P14 mice was dissected with the sciatic nerve attached. Intracellular recording was performed in oxygenated normal mammalian Ringer's solution (in mM, 135 NaCl, 5 KCl, 1 MgSO_4_, 15 NaHCO_3_, 1 Na_2_HPO_4_, 11 D-glucose, 2.5 Calcium gluconate, pH 7.4). Muscle contraction was blocked by pre-incubating the muscle in 2–3 µM μ-conotoxin (Biomol, US) for 45 minutes. The recording was then performed in the toxin-free Ringer's solution. At least 7–10 miniature endplate potentials (MEPPs) and 40–70 evoked endplate potentials (EPPs) were recorded from a given junction. The EPPs were elicited by 1 Hz train, normalized to −50 mV and corrected for nonlinear summation [67]. The mean quantal content was calculated by the direct method [25]. Synaptic transmission was also assessed by paired-pulse stimulation (10 ms apart) and high frequency (50 Hz) stimulation. The size of the readily releasable pool was estimated according to Elmqvist and Quastel [27]. The quantal content of each EPP in response to 50 Hz stimulation was plotted against the cumulative quantal content. The initial linear depression part was fitted with a linear function and extrapolated to cross the x-axis to obtain an approximate RRP size. The first EPP was not included in the linear fitting in both control and SMNΔ7 since both show initial facilitation. The probability of release was estimated by dividing the first EPP quantal content by the RRP size [28]. Data was acquired and analyzed by pClamp8 software and Minialysis software.

### Muscle Twitch tension

Muscle and nerve preparations were placed in *Sylgard* (Dow Corning, MI) coated dishes and bathed in normal Ringer's solution. The distal end of the muscle was attached to a force transducer (UC2, Gould-Statham, OH) with a nylon thread. Muscle contraction was evoked by stimulating the nerve via a suction electrode (indirect stimulation) or by directly stimulating the muscle via silver wires (direct stimulation) at 1, 10, 40 and 100 Hz. The direct stimulation was performed with the presence of d-tubocurarine (1 mM) to block the nerve evoked muscle contraction. The muscle length was adjusted to maximize the force produced. The ratio of indirect/direct stimulation evoked muscle contraction was computed and compared for SMNΔ7 and control mice.

### Light and electron microscopy of the anterior horn spinal cord

Animals were anesthetized and perfusion-fixed with 4% paraformaldehyde. Lumbar spinal cord segment (L3–5) were dissected and processed for cryosections (16 µm-thick). Motoneurons were labeled with anti-choline acetyltransferease (ChAT, *Chemicon*) antibody and presynaptic nerve terminals with anti-synaptophysin. Different excitatory synapses were labeled by anti-vesicular glutamate transporter 1 and 2 (VGLUT1 and 2) antibodies (*Synaptic System*; 1∶500). Inhibitory synapses were labeled by anti-vesicular GABA transporter (vGAT) antibody (*Synaptic System*, 1∶500). Spinal cord sections were imaged using 100× oil-immersion objectives on the Zeiss LSM confocal microscope. All confocal images were taken using the same imaging parameters (e.g. laser intensities, amplification gains and offsets). α-motoneurons were identified based on immunoreactivity to ChAT and location in the ventral horn of the spinal cord. Only large motoneurons (area>300 µm^2^) [30] with clear nuclei were selected for imaging and quantification. Selected motoneurons were imaged at optical sections of ∼0.7 µm thick showing clear cell boundaries. Immuno-labeled axosomatic synapses were identified as boutons apposed to membrane of motoneuron soma and proximal dendrites (<50 µm from soma) with no visible intervening space. The number of synaptic boutons around the motoneuron soma and proximal dendrites (identifiable with ChAT-immunoreactivity) was manually counted and normalized to the cell perimeter measured with Image J (NIH imaging software). To confirm that imaged motoneurons were devoid of necrotic/ apoptotic features, standard Hematoxylin and Eosin staining was performed on the same section after confocal imaging. Motoneurons showing necrotic/ apoptotic features e.g. eosinophilic cytoplasm, red nucleus, and apoptotic bodies were excluded in the analyses. For electron microscopy, α-motoneurons were identified by size (area>300 µm^2^) and location in the ventral horn of the spinal cord. Synapses were identified as boutons enclosing synaptic vesicles and having electron-dense postsynaptic density. Synaptic bouton number, apposition length and motoneuron perimeter were measured with Image J (NIH imaging software). To visualize the association of motoneurons with microglia, cryosections of the lumbar (L3–5) spinal cord were immuno-labeled with anti-Iba1 antibody (Wako chemical; 1∶500) for microglia and anti-ChAT for motoneurons.

### Quantification of proprioceptive dorsal root axons and ganglion

Dorsal root axons from L4 spinal segment were dissected, post-fixed in osmium tetroxide and subsequently embedded in Epon. One µm-thick sections of the axons were stained with toluidine blue. The number and size of the myelinated axons were counted using Image J. To examine the number of proprioceptive sensory neurons, L4 dorsal root ganglion (DRG) were prepared for cryosections (16 µm-thick). Every fifth serial section was used for immuno-staining of parvalbumin (PV), a marker for proprioceptive sensory neurons [34]. The total number of PV-positive neurons in each DRG was calculated by multiplying the average number of PV-positive cells per section to the total number of sections collected from sectioning through the entire DRG.

### Statistical analyses

Data was statistically analyzed using two-tailed Student's *t-tests* with statistic software (Prism 5.0). *p*<0.05 was considered significant. Results were expressed as mean ± s.e.m.

## Supporting Information

Figure S1 **Perisynaptic Schwann cells are co-localized with nerve-muscle contacts in both fast and slow muscle types.** The presence of perisynaptic Schwann cells (labeled with anti-S100 antibody) at NMJ were quantified as “innervated NMJ” in a fast muscle extensor digitorum longus (EDL) and a slow muscle soleus (SOL) (EDL: Control, 99.4±0.4%, n = 761 NMJs in 3 animals; SMNΔ7 SMA, 99.4±0.2%, n = 484 NMJs in 2 animals; SOL: Control, 99.6±0.4%, n = 675 NMJs in 3 animals; SMNΔ7 SMA, 95.9±2.0%, 414 NMJs in 3 animals). (TIF)

Figure S2 **Accumulation of neurofilament at the SMNΔ7 NMJs at the end-stage.** Neurofilaments labeled with anti-neurofilament antibody (in green) was observed in the majority of nerve terminals at neuromuscular junctions (labeled with α-bungarotoxin, in red) in the gastrocnemius muscle of SMNΔ7 mice at P14. (TIF)

Figure S3 **SMNΔ7 lateral motoneurons in the L3–L5 spinal segments morphologically resemble control motoneurons.** Spinal cord sections of the control and SMA mice were immuno-stained with anti-synaptophysin (green) for synapses and anti-ChAT for motoneurons (red) (A & C). After quantification of synapses, the same sections were processed with Hematoxylin and Eosin staining (B & D). SMNΔ7 motoneurons morphologically resembled control motoneurons and did not show chromatolytic or apoptotic changes. (TIF)

Figure S4 **A size analysis of synapse onto L3–L5 lateral motoneurons in SMNΔ7 mice at the end-stage.** Average bouton size on L3–L5 lateral motoneurons in control and SMNΔ7 SMA mice are similar despite a ∼34% reduction in the size of postsynaptic density (PSD) normalized to presynaptic bouton size. (A) Bar graph showing the average bouton size on motoneurons in control and SMNΔ7 SMA mice (Control, 1.26±0.05 µm, n = 162 boutons; SMNΔ7, 1.31±0.06 µm, n = 160 boutons, *p* = 0.46). (B) Bar graphs showing the percentage of PSD length relative to the synaptic length (Control, 22.0±2.0%, n = 10 motoneurons; SMN7, 14.6±1.9%, n = 15 motoneurons; *p* = 0.014). (TIF)

Figure S5 **Glutamatergic synapses on SMNΔ7 motoneurons in L3–L5 spinal segments are not reduced at P7.** The number of VGLUT1 and VGLUT2 synapses on L3–L5 lateral motoneurons in control and SMNΔ7 mice is similar at P7. VGLUT1, 5.1±0.3 (n = 65 motoneurons) vs. 5.4±0.8 (n = 30 motoneurons) and VGLUT2, 32.4±3.2 (n = 31 motoneurons) vs. 30.3±10.8 (n = 36 motoneurons). (TIF)
