# Supplementary figures and images for: Synaptic Defects in the Spinal and Neuromuscular Circuitry in a Mouse Model of Spinal Muscular Atrophy
Source: PLoS One. 2010 Nov 11;5(11):e15457. doi: 10.1371/journal.pone.0015457 (PMC2978709; doi:10.1371/journal.pone.0015457)

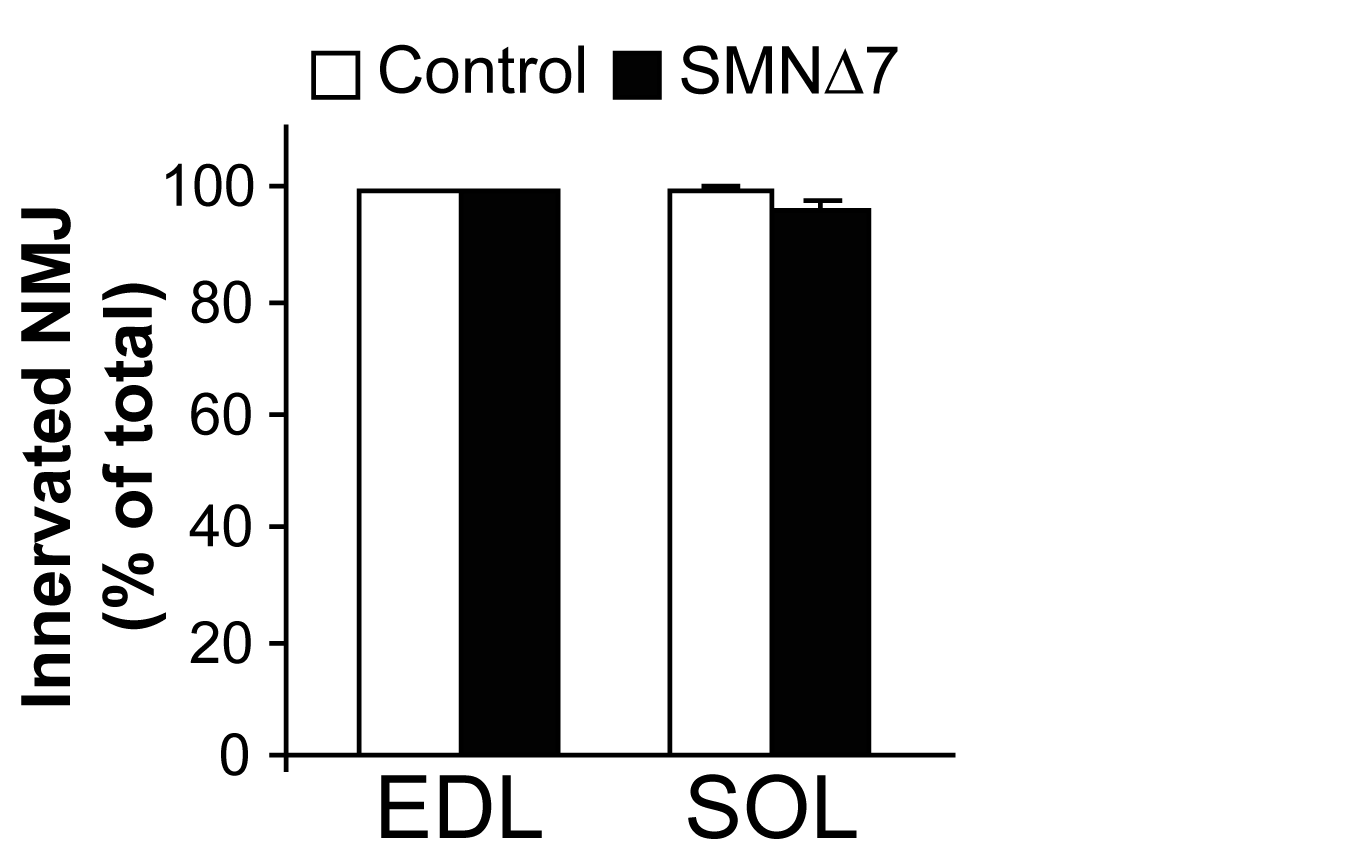

Supplement: Figure S1 — Perisynaptic Schwann cells are co-localized with nerve-muscle contacts in both fast and slow muscle types. The presence of perisynaptic Schwann cells (labeled with anti-S100 antibody) at NMJ were quantified as “innervated NMJ” in a fast muscle extensor digitorum longus (EDL) and a slow muscle soleus (SOL) (EDL: Control, 99.4±0.4%, n = 761 NMJs in 3 animals; SMNΔ7 SMA, 99.4±0.2%, n = 484 NMJs in 2 animals; SOL: Control, 99.6±0.4%, n = 675 NMJs in 3 animals; SMNΔ7 SMA, 95.9±2.0%, 414 NMJs in 3 animals). (TIF) [file pone.0015457.s001.tif]

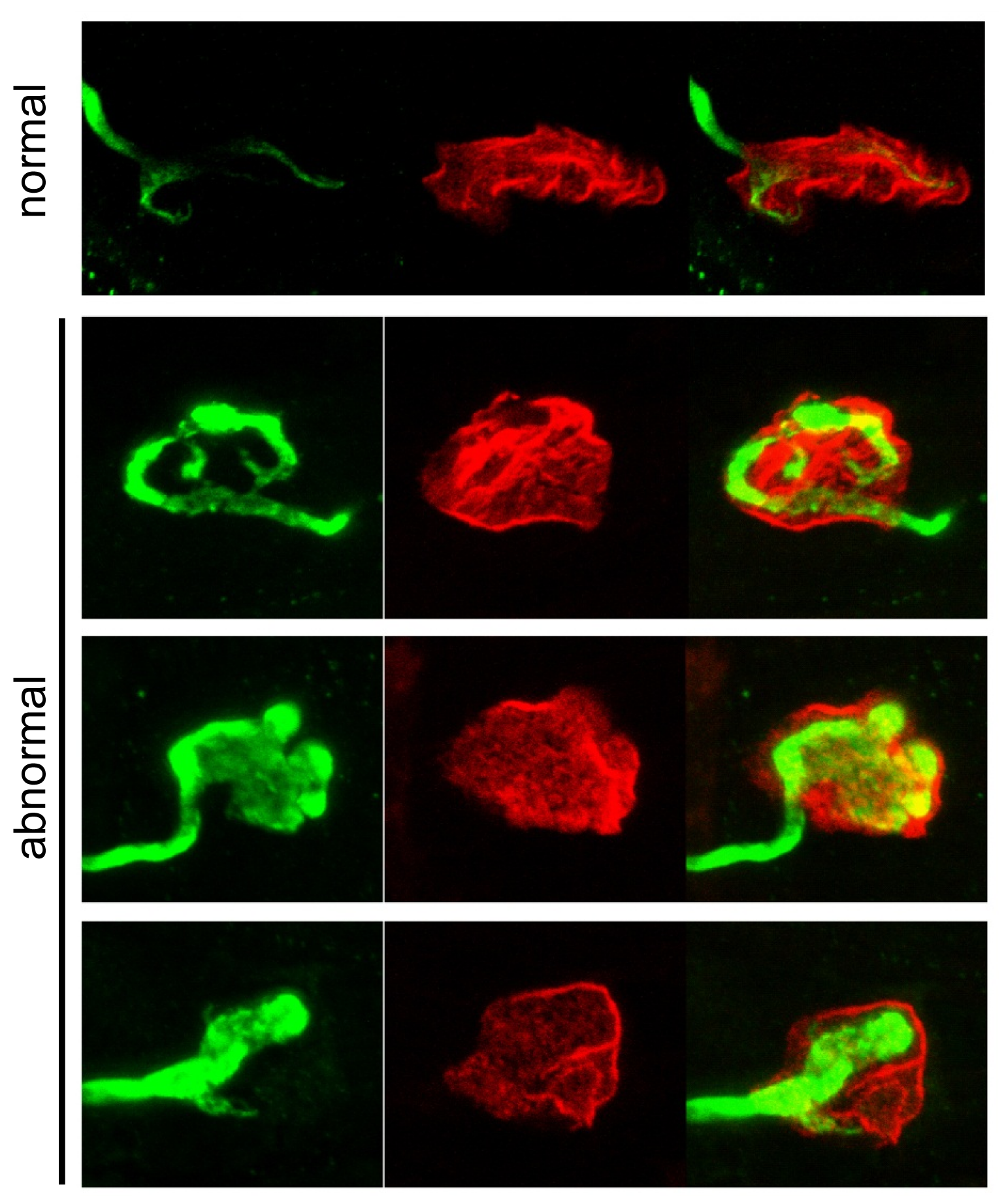

Supplement: Figure S2 — Accumulation of neurofilament at the SMNΔ7 NMJs at the end-stage. Neurofilaments labeled with anti-neurofilament antibody (in green) was observed in the majority of nerve terminals at neuromuscular junctions (labeled with α-bungarotoxin, in red) in the gastrocnemius muscle of SMNΔ7 mice at P14. (TIF) [file pone.0015457.s002.tif]

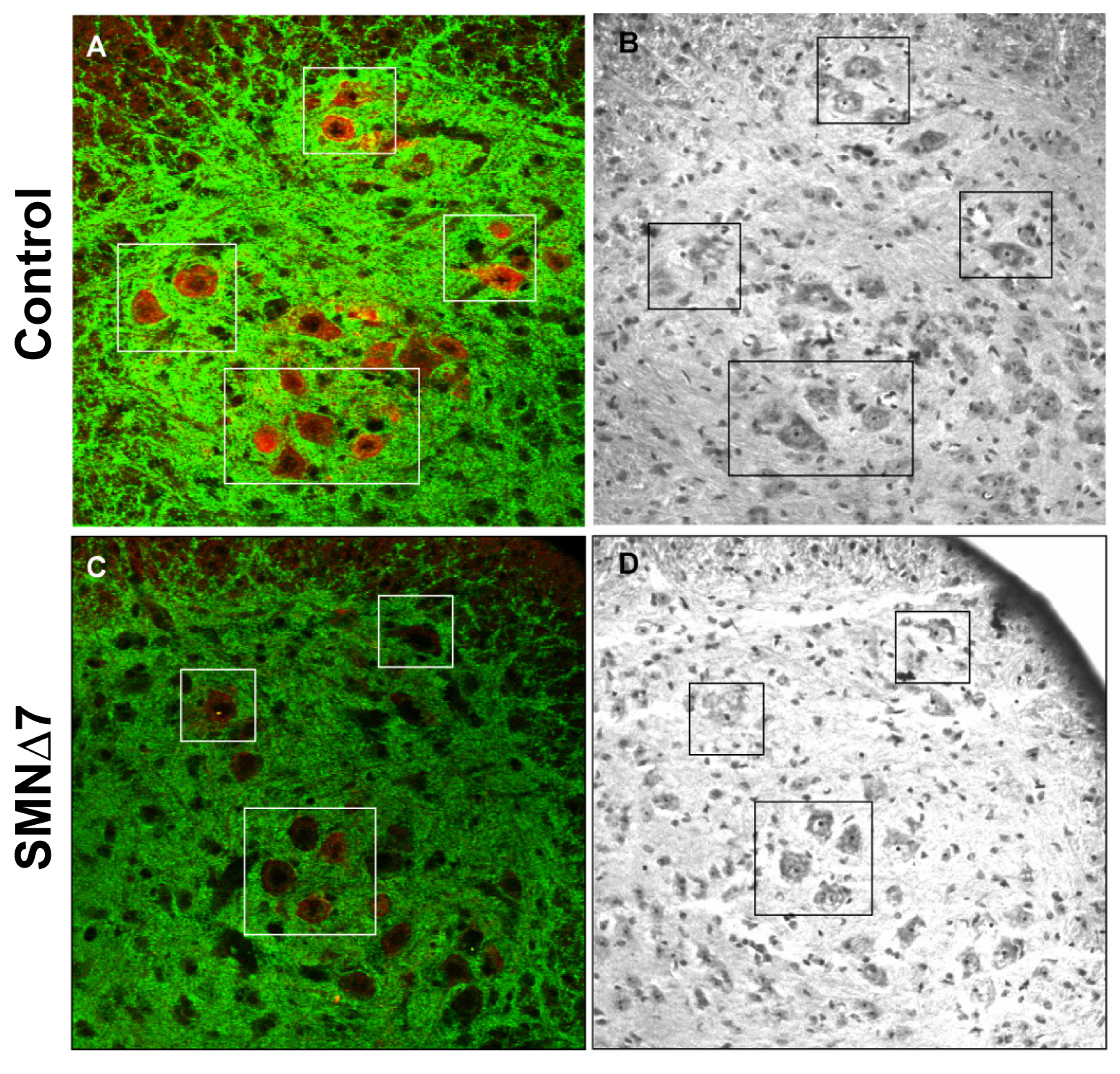

Supplement: Figure S3 — SMNΔ7 lateral motoneurons in the L3–L5 spinal segments morphologically resemble control motoneurons. Spinal cord sections of the control and SMA mice were immuno-stained with anti-synaptophysin (green) for synapses and anti-ChAT for motoneurons (red) (A & C). After quantification of synapses, the same sections were processed with Hematoxylin and Eosin staining (B & D). SMNΔ7 motoneurons morphologically resembled control motoneurons and did not show chromatolytic or apoptotic changes. (TIF) [file pone.0015457.s003.tif]

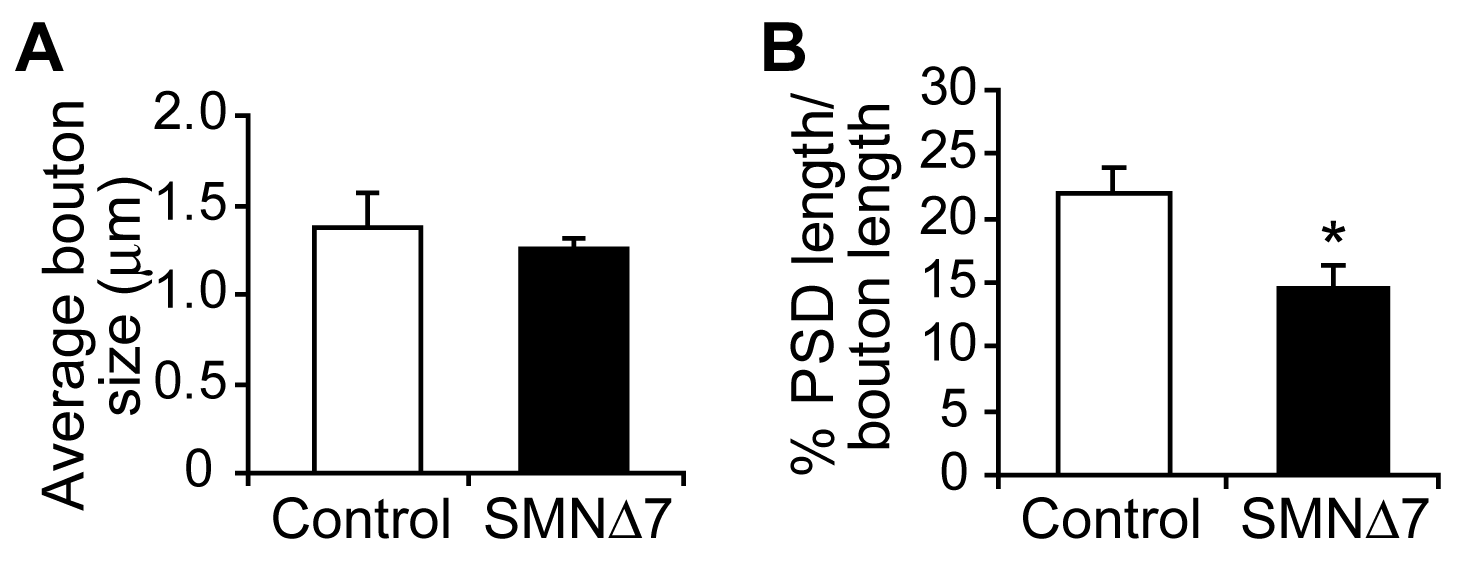

Supplement: Figure S4 — A size analysis of synapse onto L3–L5 lateral motoneurons in SMNΔ7 mice at the end-stage. Average bouton size on L3–L5 lateral motoneurons in control and SMNΔ7 SMA mice are similar despite a ∼34% reduction in the size of postsynaptic density (PSD) normalized to presynaptic bouton size. (A) Bar graph showing the average bouton size on motoneurons in control and SMNΔ7 SMA mice (Control, 1.26±0.05 µm, n = 162 boutons; SMNΔ7, 1.31±0.06 µm, n = 160 boutons, p = 0.46). (B) Bar graphs showing the percentage of PSD length relative to the synaptic length (Control, 22.0±2.0%, n = 10 motoneurons; SMN7, 14.6±1.9%, n = 15 motoneurons; p = 0.014). (TIF) [file pone.0015457.s004.tif]

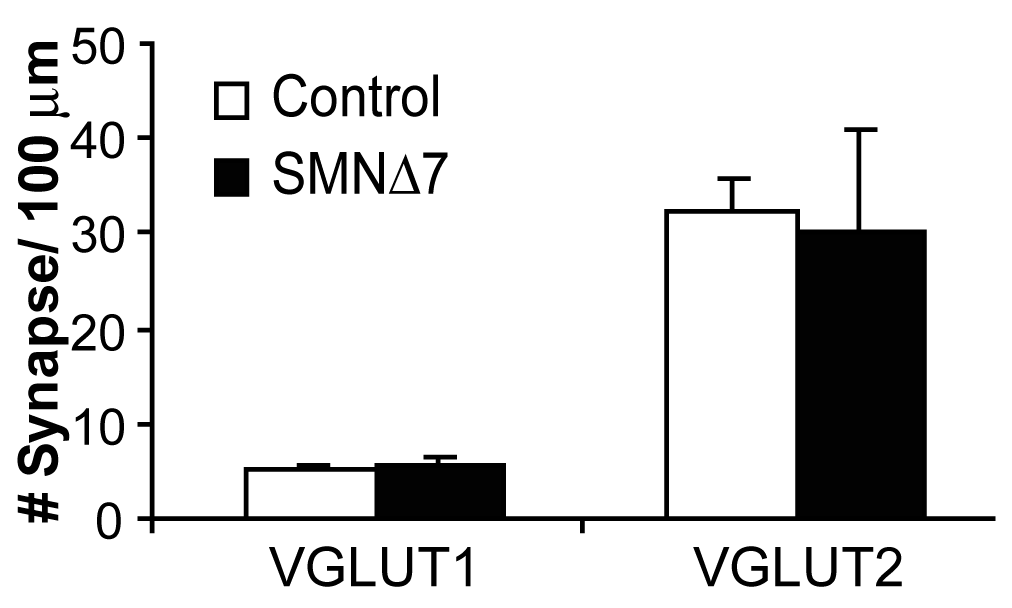

Supplement: Figure S5 — Glutamatergic synapses on SMNΔ7 motoneurons in L3–L5 spinal segments are not reduced at P7. The number of VGLUT1 and VGLUT2 synapses on L3–L5 lateral motoneurons in control and SMNΔ7 mice is similar at P7. VGLUT1, 5.1±0.3 (n = 65 motoneurons) vs. 5.4±0.8 (n = 30 motoneurons) and VGLUT2, 32.4±3.2 (n = 31 motoneurons) vs. 30.3±10.8 (n = 36 motoneurons). (TIF) [file pone.0015457.s005.tif]
